# What should medical students be taught about abortion? An evaluation of student attitudes towards their abortion teaching and their future involvement in abortion care

**DOI:** 10.1186/s12909-020-02414-9

**Published:** 2021-01-04

**Authors:** Pollyanna Cohen, Jonathan Mayhew, Faye Gishen, Henry W. W. Potts, Patricia A. Lohr, Jayne Kavanagh

**Affiliations:** 1 University College London Medical School, London, UK; 2 Oxford University Hospitals NHS Foundation Trust, Oxford, UK; 3 Institute of Health Informatics, University College London, London, UK; 4 British Pregnancy Advisory Service (BPAS), London, UK

**Keywords:** Abortion, Medical students, Women’s health, Ethics and law, Inclusive curriculum

## Abstract

**Background:**

One in three women in the United Kingdom (UK) will have an abortion before age 45, making abortion provision an essential aspect of reproductive healthcare. Despite this, abortion remains ethically contested and stigmatised, with variable teaching in UK medical schools and concerns about falling numbers of doctors willing to participate in abortion care. University College London Medical School (UCLMS) has designed practical, inclusive, teaching that aims to give students an understanding of the importance of abortion care and prepare them to be competent practitioners in this area. This study aimed to determine students’ opinions of this teaching and their wider attitudes towards abortion.

**Methods:**

We invited all 357 final-year UCL medical students to complete an online survey consisting of closed-ended questions, exploring their opinions on their abortion teaching, their personal beliefs about abortion and their future willingness to be involved in abortion care. We analysed responses using non-parametric tests.

**Results:**

One hundred and forty-six questionnaires (41% response rate) showed 83% of students identified as pro-choice (agree with the right to choose an abortion). Fifty-seven percent felt they received the right amount of abortion teaching, 39% would have liked more and 4% stated they received too much. There was no correlation between students’ attitudes to abortion and the rating of teaching; both pro-choice and pro-life (opposed to the right to choose an abortion) students generally rated the teaching as important and valued the range of methods used. Students requested more simulated practice speaking to patients requesting an abortion. Students with pro-life beliefs expressed lower willingness to discuss, refer, certify and provide future abortions. Students interested in careers in specialties where they may encounter abortion were more likely to be pro-choice than pro-life.

**Conclusions:**

The majority of participating UCL medical students were pro-choice and willing to be involved in future abortion care. Efforts to make teaching on abortion practical, engaging, sensitive and inclusive were appreciated. As well as preparing students to be competent and caring practitioners, the teaching appears to contribute towards them viewing abortion as an essential aspect of women’s healthcare, and may contribute to destigmatising abortion.

**Supplementary Information:**

The online version contains supplementary material available at 10.1186/s12909-020-02414-9.

## Background

One in three UK women will have an abortion by the age of 45 [1], making abortion provision an essential and common aspect of reproductive healthcare. Despite this, abortion remains an ethically contested and stigmatised issue both for those who have them and for those who provide them [2]. This is likely to be a significant factor in the decreasing number of junior doctors interested in providing abortions in the UK; only 33 Obstetrics and Gynaecology (O&G) trainees have completed the Royal College of Obstetricians and Gynaecologists (RCOG) specialist abortion care training since 2007 and only 20 clinicians have undertaken the Faculty of Sexual and Reproductive Health’s (FSRH) abortion care module in that time. The low numbers of clinicians interested in specialist training has led to concerns about a future lack of abortion providers [3].

The RCOG has highlighted the insufficient and variable educational opportunities in abortion care for both medical students and O&G trainees for a number of years [4], emphasising the importance for medical students of gaining an understanding of patients’ experiences of abortion and the significance of abortion to their future practice [3]. Previous UK studies found that 62% [5] and 73% [6] of UK medical students identified as pro-choice (agree with the right to choose an abortion). Medical students also recognise the importance of including abortion education in the medical school curriculum [6]. Yet evidence suggests that abortion education lacks clinical content, including explanations of specific procedures, as well as exposure to direct patient care in a number of UK medical schools [7].

The RCOG’s national undergraduate curriculum provides guidance for medical schools on abortion teaching [8]. Students are expected to be able to understand and demonstrate appropriate knowledge, skills and attitudes in relation to abortion. This includes an understanding of the clinically relevant aspects of the legal framework for abortion in the UK, the 1967 Abortion Act, the need to respect religious and cultural beliefs, knowledge of methods, indications, contraindications and complications of abortion as well as the ability to take an abortion-related history. The General Medical Council (GMC), who provide the professional framework for all undergraduate medical education in their Outcomes for Graduates [9], specify students should ‘recognise the potential impact of their attitudes, values, beliefs, perceptions and personal biases (which may be unconscious) on individuals and groups and identify personal strategies to address this’. Furthermore, the RCOG acknowledges that some students may hold a conscientious objection to abortion, but state that it is important that they have an understanding of abortion care and are competent to provide appropriate emergency care in their future careers [8].

In line with the RCOG’s guidance on curriculum content and the competencies needed to practice as a Foundation Year doctor, abortion teaching at University College London Medical School (UCLMS) aims to prepare students, including those with a conscientious objection, to practice in a clinically, legally, professionally and ethically robust way when they see a person who requests an abortion or has an abortion-related complication (Table 1).

**Table 1:** UCLMS teaching framework for developing clinically, legally and ethically competent junior doctors

Legal	Abortion law, conscientious objection, abortion (HSA1) form
Clinical	Methods, complications, history, support, safeguarding
Professional and Ethical	Personal beliefs, opting out, stigma, language, behaviour, professional role

In order to assess our teaching and explore UCLMS medical students’ personal beliefs about abortion, we reviewed the literature to find out what’s already known about the teaching of abortion to medical students, and we devised a questionnaire to capture students’ views on:

- The strengths of our abortion teaching and how it could be improved.
- How their personal values shaped their experience of the teaching.
- How their personal beliefs might affect their future involvement in abortion care.

## Methods

### Setting and participants

UCLMS uses a range of teaching methods to engage students in two dedicated half day sessions on abortion in the medical ethics and law (Year 2) and in the women’s health programmes (Year 5) of the core undergraduate medical curriculum. The Year 2 session focuses on the legal, ethical and political aspects of abortion. The Year 5 (penultimate year) session focuses on what students need to know to be competent Foundation Year 1 doctors, including the clinical aspects of abortion care, the practicalities of consultations, analyses of reasons for abortion, value-laden language, counteracting stigma, and conscience and abortion provision. Students can also opt-in to a half-day clinical placement in abortion care in Year 5. Only 38% of UK medical schools provide this opportunity [10]. Additionally, interactions with doctors with a conscientious objection and doctors with a conscientious commitment to abortion care in both Years 2 and 5, as well as with a woman who has had an abortion in Year 5, contribute to an inclusive, practical and patient-focused learning experience.

We surveyed all final year UCL MBBS Medicine students who had completed their abortion teaching at UCLMS in November 2017. Participants were informed that the research results would be used to improve UCLMS abortion teaching.

### Survey generation

We devised an online questionnaire: see Additional file 1. We did not find an existing appropriate validated survey, but drew on existing similar studies of medical students in our design [5, 6, 11, 12]. The questionnaire was approved by medical education professionals, abortion researchers and a medical student from the participant cohort. We used closed-ended questions with binary response options, multiple responses or Likert scales, exploring students’ opinions about their education on abortion, their beliefs about abortion and their involvement in abortion care in the future. They were also given the opportunity to provide free text responses on their experience of their abortion education throughout.

### Survey implementation

UCL email addresses were obtained through UCL Medical School’s database and emails were sent to all final year UCL medical students. The email had a link to an information sheet and invited them to voluntarily complete the online questionnaire. Data was gathered through Opinio, a secure online survey tool that anonymises raw data. Two further reminder emails were sent to students, one week apart, to maximise the response rate. As the study did not collect any personally-identifying information we were unable to send targeted reminder emails to non-responders. The Opinio report was stored, accessed and analysed through a password-protected account.

Ethics permission was granted via UCL Research Ethics Committee (Number 4415/003).

### Statistical analyses

We analysed responses using Kruskal-Wallis and Friedman statistical tests and Spearman’s correlations. We explored the relationship between students’ religion and opinion, performing a Kruskal-Wallis test treating overall abortion attitude as an ordinal variable, followed by post hoc Mann-Whitney tests to determine which groups differed from which.

Students were asked about their future involvement in abortion care. They were asked about their willingness to discuss, refer, complete an HSA1 form, or provide abortions at less than 12 weeks’ gestation, 12–24 weeks’ gestation and over 24 weeks’ gestation. A Friedman test was conducted to assess whether students’ willingness to participate in abortion care decreased as the level of involvement increased (from referring patients to providing abortions) or as gestation increased. Themes from free-text comments were summarised.

## Results

### Response rate and demographics

146 of 357 (41%) final year students completed some or all of the questionnaire. Mean age was 24, see Table 2 for other participant demographics.

**Table 2:** Demographics and attitudes towards abortion of UCLMS final-year medical students

**Gender, n(%)**	
Male	27(27%)
Female	68(69%)
Non-binary	1(1%)
Prefer not to say	3(3%)
TOTAL	99
**Attitude to abortion, n(%)**	
Pro-choice	84(83%)
Pro-life	13(13%)
Neither	2(2%)
Undecided	1(1%)
Prefer not to say	1(1%)
TOTAL	101
**Religion, n(%)**	
Yes	50(51%)
No	36(36%)
Other/Prefer not to say	13(13%)
TOTAL	99

Regarding religion, the Kruskal-Wallis test treating overall abortion attitude as an ordinal variable was statistically significant: χ^2^(4) = 16.1, *p* = 0.003. The no religion group was statistically significantly more pro-choice than the Protestant (post hoc Mann-Whitney test: *p* = 0.039) or Muslim (*p* = 0.0001) groups. However, the Catholic group (*p* = 0.2) were neither more nor less pro-choice compared to the no religion group.

### Perceptions of the UCLMS abortion teaching

103 of 107 (96%; 95% confidence interval: 91–99%) students rated their abortion teaching as ‘somewhat’ or ‘very important’. There was no correlation between whether students identified as pro-choice or pro-life and their rating of the importance of teaching (Spearman’s correlation = − 0.0, *p* = 0.7).

57% of students felt the amount of teaching was ‘about right’, 39% felt there was ‘too little’ teaching and 4% felt there was ‘too much’ teaching (Table 3).

**Table 3:** Opinions on amount of teaching of individual topics

	Much too little	A bit too little	About right	A bit too much	Much too much	Total
**UK law on abortion**	1 (1%)	11 (10%)	97 (89%)	0 (0%)	0 (0%)	109
**Doctors’ legal and professional right to opt out of providing abortion care**	3 (3%)	12 (11%)	80 (73%)	12 (11%)	2 (2%)	109
**Conducting a pregnancy options consultation**	18 (17%)	59 (54%)	30 (28%)	2 (2%)	0 (0%)	109
**How to respectfully opt out of abortion care**	9 (8%)	26 (24%)	61 (56%)	10 (9%)	3 (3%)	109
**Identifying women who may need counselling before making a decision about abortion**	23 (21%)	52 (48%)	32 (29%)	2 (2%)	0 (0%)	109
**Supporting women to make a decision about abortion**	15 (14%)	48 (44%)	44 (40%)	1 (1%)	1 (1%)	109
**Referring to an abortion provider**	14 (13%)	40 (37%)	53 (49%)	2 (2%)	0 (0%)	109
**Completing the HSA1 (abortion) form**	11 (10%)	21 (19%)	75 (69%)	0 (0%)	1 (1%)	108
**What surgical abortion (vacuum aspiration) entails**	5 (5%)	39 (36%)	63 (58%)	2 (2%)	0 (0%)	109
**What a surgical abortion (dilatation and evacuation) entails**	5 (5%)	42 (39%)	59 (54%)	3 (3%)	0 (0%)	109
**What early medical abortion (< 10 weeks gestation) entails**	2 (2%)	34 (31%)	70 (64%)	3 (3%)	0 (0%)	109
**What later medical abortion (> 10 weeks gestation) entails**	5 (5%)	39 (36%)	63 (58%)	2 (2%)	0 (0%)	109
**Abortion-related complications/risks**	7 (6%	29 (27%)	69 (64%)	3 (3%)	0 (0%)	108
**Why women seek abortions**	10 (9%)	28 (26%)	70 (65%)	0 (0%)	0 (0%)	108
**Guest speaker: woman who has had abortion**	8 (7%)	16 (15%)	75 (69%)	7 (6%)	3 (3%)	109
**Guest speaker: doctor with a conscientious objection to abortion**	17 (16%)	24 (22%)	56 (51%)	9 (8%)	3 (3%)	109
**Clinical exposure to abortion provision**	29 (27%)	33 (30%)	46 (42%)	1 (1%)	0 (0%)	109
**Moral arguments for and against abortion**	6 (6%)	20 (18%)	69 (63%)	9 (8%)	5 (5%)	109
**TOTAL**	**188 (10%)**	**573 (29%)**	**1112 (57%)**	**68 (3%)**	**18 (1%)**	

Students’ opinions were divided about attendance at abortion clinics (Table 4).

**Table 4:** Responses to: Should attendance at an abortion clinic be optional or compulsory?

	n(%)	95% Confidence Interval
**Optional**	44(43%)	33–53%
**Compulsory**	46(45%)	35–55%
**Unsure**	12(12%)	6–20%
Total	102	

The majority of free text comments were encouraging (Table 5).

**Table 5:** Examples of free text comments about teaching

Comment	Attitude
This module has been run in a very well structured as well as exceptionally professional and sensitive manner.	Pro-life
I really value the time we dedicate to learning and discussing medical ethics issues, I think these experiences are part of what will make us empathetic, intelligent, resilient doctors.	Pro-choice

Eight students described the teaching as ‘excellent’, while another eight wanted more simulated practice speaking to women requesting an abortion. Seven students mentioned the teaching on conscientious objection, with opposing opinions depending on their beliefs. Four pro-choice students felt there was ‘too much emphasis’ on conscientious objection, whereas three pro-life students felt there was ‘not much teaching on conscientious objection’.

### Potential future involvement in abortion care

Students’ willingness to participate in abortion care decreased significantly as the level of involvement increased (from referring patients to performing abortions), for all gestations (< 12 weeks [W = 0.33, *p* = 0.0001], 12–24 weeks [W = 0.35, *p* < 0.0001], and at > 24 weeks [W = 0.48, *p* < 0.0001]) (Table 6). Students’ attitude to abortion was correlated with their willingness to carry out different levels of care. All relationships were statistically significant – as students’ attitudes became more pro-life, students became less willing to discuss, refer, complete an HSA1 (abortion) form or provide a medical or surgical abortion (*p* < 0.05 using Spearman’s correlation). The correlations became stronger with increased involvement in care.

**Table 6:** Students’ willingness to engage in different levels of abortion care at different gestations

	Total number of students (*N* = 102) who said “yes, in any legally justifiable circumstances” or “yes, but in specific circumstances only”					
	Gestation					
	< 12 weeks,		12–24 weeks		> 24 weeks	
	n(%)	95% Confidence Interval	n(%)	95% Confidence Interval	n(%)	95% Confidence Interval
Level of involvement						
Pregnancy options discussion	99(97%)	92–99%	99(97%)	92–99%	92(90%)	83–95%
Refer for an abortion	96(94%)	88–98%	95(93%)	86–97%	89(87%)	79–93%
Complete HSA1 (abortion) form	91(89%)	82–94%	90(88%)	80–94%	85(83%)	75–90%
Provide medical abortion	74(73%)	63–81%	70(69%)	59–77%	58(57%)	47–67%
Provide surgical abortion	65(64%)	54–73%	62(61%)	51–70%	52(51%)	41–61%

**Fig. 1:**
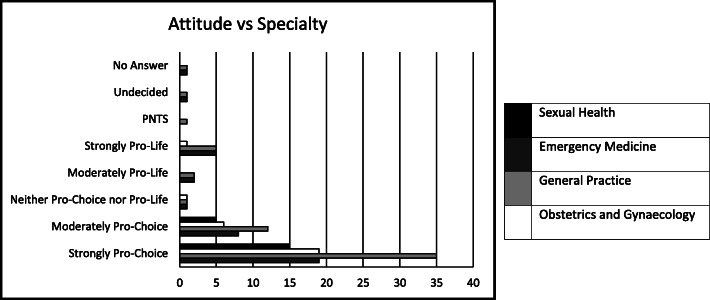
Students’ attitudes versus their future specialty interest

More pro-choice students (119) than pro-life students (15) were interested in specialties where they may encounter abortion care; 30% of pro-choice students were interested in a career in O&G versus 8% of pro-life students (Fig. 1).

## Discussion

We found that among UCLMS final year students, who had seven hours of abortion teaching integrated into their curriculum, a higher number (83%) were pro-choice than previous research which reported percentages as low as 16.3% in 2009 [11]. Gleesen et al. [5] suggested that attitudes among medical professionals could be becoming more pro-choice and our findings support this. Similarly, a 2019 survey of Glasgow medical students showed that 83% identified as pro-choice following teaching based on the UCLMS materials [13]. Over the last few years, there seems to be an ever-growing attitude shift among healthcare professionals towards accessible and safe abortion care with several professional bodies including the RCOG, the FSRH, the Royal College of General Practitioners, the Royal College of Nursing, the Royal College of Midwives and the British Medical Association all supporting the decriminalisation of abortion.

Overall, most students felt they received the right amount of abortion teaching and fewer wanted more compared to previous studies – 39% versus over 50% of students in Oldroyd’s study who felt they ‘had not received enough’ teaching [10]. A similarly high number of students (96%) rated abortion teaching as important compared to previous studies (95% [6]) and, for UCLMS students, this was irrespective of whether they identified as pro-choice or pro-life. Students’ positive responses to the UCLMS teaching, their requesting more reminders for clinics and simulated practice of abortion consultations, demonstrates their appetite for thorough, practical teaching which prepares them to be competent, respectful and compassionate practitioners, regardless of their attitudes towards abortion. It is possible that the lack of correlation between students’ attitudes to abortion and their rating of the importance of teaching also reflects the efforts made by teaching faculty to not only ensure teaching is inclusive and respectful of all beliefs, but also to make it engaging, employing multiple changes of stimuli with guest speakers, videos, quizzes, electronic voting, discussion and simulated practice embedded in the more traditional lecture and small group format.

Our study showed similar findings to others with regard to religion – small numbers in each religious group meant we were unable to statistically compare students’ attitudes towards abortion with their beliefs [5]. Another result consistent with other studies was that UCLMS students were less likely to be willing to be involved in providing medical or surgical abortion at later, rather than earlier, gestations [5].

Students interested in specialties potentially involving abortion care, such as O&G, Sexual Health, Emergency Medicine and General Practice, were more likely to be pro-choice than pro-life. Considering the concern about a future lack of abortion care providers, waning interest in O&G training and the prevalence of abortion itself, this was reassuring. It also supports the argument that the lack of uptake of specialist RCOG and FSRH training in abortion care is not simply a case of pro-life trainees opting out, but encompasses other factors such as the practical aspects of training, abortion-related stigma, workload and other life choices.

Recent research on abortion-related stigma states that ‘work remains to be done to dismantle abortion negativity embedded in the healthcare system’ [2]. The UCLMS teaching aims to counteract this negativity and abortion-related stigma in a number of ways. Firstly, by including teaching about how to counteract stigma in abortion consultations. Secondly, drawing on Allport’s intergroup contact theory approach [14], by exposing students to doctors with a conscientious commitment to abortion care, as well as doctors with a conscientious objection, and to women who have had abortions. Thirdly, by facilitators striving to create a respectful and safe environment where students are able to express their beliefs, concerns and fears. And lastly, by allocating seven hours of core curriculum time to abortion teaching and including questions on the content of this teaching in all relevant formative and summative assessments, demonstrating to students that abortion is an essential aspect of reproductive healthcare and that UCLMS is committed to preparing them to be competent practitioners in this area.

### Addressing findings

Following this study, we have reviewed and developed our teaching, adding a simulated practice exercise with a woman requesting an abortion, the anonymous story of a medical student’s abortion and further discussion on how to counteract abortion-related stigma and facilitate trust in an abortion consultation, including identifying and avoiding value-laden language. We have also refined our material on the difference between conscientious objection and obstructing abortion care, as well as emphasising the importance of both knowing how to respectfully opt out of abortion care. To ensure sessions are inclusive and respectful of a spectrum of beliefs about abortion, we have taken steps to secure both pro-choice and pro-life speakers for each session. Finally, we are sending students follow up emails reminding them they can opt into attending abortion assessment clinic placements.

There are limited data about what abortion teaching is included in UK undergraduate healthcare professional curricula despite the prevalence of abortion. We are therefore conducting further research on the extent to which abortion education features in UK medical schools’ curricula, their aims, outcomes and content of abortion education and how it is delivered, as well as the barriers to including comprehensive abortion education in undergraduate curricula.

Furthermore, to support efforts to integrate inclusive and comprehensive abortion teaching into other undergraduate healthcare professional curricula we have established a repository for sharing our teaching resources [15].

### Limitations and future implications

The response rate of 41%, although not as low as some previous surveys of medical students’ opinions on abortion [16], was the main limitation of the study, as it may indicate a response bias where students who chose to answer the survey had the strongest opinions and so it is difficult to be sure that our sample is valid and reliable. Nevertheless, we maximised this rate with reminder emails and by surveying students months before their final examinations. We tried to avoid bias in the survey questions and encourage honest responses by assuring students there were no ‘right’ or ‘wrong’ answers, using the well-recognised terms of ‘pro-choice’ and ‘pro-life’ and allowing them to opt out of any part of the survey. Our study only represents students’ attitudes at one medical school, however the use of an online questionnaire gives the potential for this study to be reproduced at other medical schools.

## Conclusion

The majority of UCL medical students who responded to our survey were pro-choice, willing to be involved in abortion care in their future careers, wanted teaching on abortion and valued it, regardless of their personal beliefs about abortion. Efforts to make teaching on abortion practical, engaging, sensitive, inclusive and patient-focused seem to be appreciated by students, contribute towards them viewing abortion as an essential aspect of women’s health, prepare them to be competent and caring practitioners and most likely contribute to destigmatising abortion.

Providing comprehensive and effective teaching on abortion in medical schools can be challenging. It requires adequate curriculum time and expert, dedicated facilitators with knowledge of the legal, professional and ethical aspects of abortion as well as relevant clinical and teaching experience However, it is vital that medical schools take up this challenge to ensure future doctors develop the knowledge, skills and attitudes to care for patients competently and with respect whether they wish to opt out of abortion care or not, as required by the GMC, BMA and RCOG.

## Supplementary Information

**Additional file 1.** Survey questions.

## Data Availability

Resources used for UCLMS abortion teaching (including session plan and PowerPoint slides) https://www.doctorsforchoiceuk.com/undergradteaching The datasets generated and/or analysed during the current study are available from the corresponding author on reasonable request.

## Abbreviations

BMA: British Medical Association
FSRH: Faculty of Sexual and Reproductive Healthcare
GMC: General Medical Council
O&G: Obstetrics and Gynaecology
RCOG: Royal College of Obstetrics and Gynaecology
UCL: University College London
UCLMS: University College London Medical School
