# Supplementary material for: What should medical students be taught about abortion? An evaluation of student attitudes towards their abortion teaching and their future involvement in abortion care
Source: BMC Med Educ. 2021 Jan 4;21:4. doi: 10.1186/s12909-020-02414-9 (PMC7784357; doi:10.1186/s12909-020-02414-9)
Supplement: Supplementary file 1 — Additional file 1. Survey questions. [file 12909_2020_2414_MOESM1_ESM.pdf]

Abortion Education at UCL Medical School: Anonymous Research Survey

This survey asks about your knowledge and opinions on abortion care and abortion education at UCLMS.

There are no right or wrong answers; the survey is entirely anonymous. Please answer as honestly as you can.

Your knowledge of abortion care

1. In general, how good or poor is your knowledge about the following topics?

	Very poor	*Somewhat poor*	*Neither poor nor good*	*Somewhat good*	*Very good*
UK law on abortion.					
Doctors' legal and professional right to opt out of providing abortion care.					
Conducting a pregnancy options consultation.					
How to respectfully opt out of abortion care.					
Identifying women who may need counselling before making a decision about abortion.					
Supporting women to make a decision about abortion.					
Referring to an abortion provider.					
Completing a HSA1 (abortion) form.					
What surgical abortion (vacuum aspiration) entails.					
What surgical abortion (dilatation and evacuation) entails.					
What early (<10 weeks gestation) medical abortion entails.					
What late (>10 weeks gestation) medical abortion entails.					
What the main abortion-related complications /risks are.					
Why women seek abortions.					

Moral arguments for and against abortion.					

Your teaching on abortion at UCL Medical School

3. At UCLMS have you had a voluntary placement in an abortion assessment clinic? (please circle)
No / Yes

If YES to Q3, How much do you agree or disagree that your experience helped you…

	Strongly Disagree	*Disagree*	*Neither agree nor disagree*	*Agree*	*Strongly Agree*
…understand how abortion assessment consultations are conducted					
…understand why women seek abortions					
…think more clearly about my own involvement in abortion care					

If NO to Q3, why did you not take part in an abortion assessment clinical placement? (please tick all that apply)

I didn't think it would be useful	☐
I have a conscientious objection to abortion	☐
I didn't know about the placements	☐
I wanted to but there were no placements available	☐
I booked a placement but it was cancelled by the clinic	☐
I booked a placement but it I had to cancel it	☐

Other (please write): ______________________________

4. Have you received any clinical teaching on abortion outside of Year 2 and Year 5 Ethics and Law teaching, within your clinical placements? (please circle) No / Yes

If YES, please specify which aspects of abortion care which were covered:

5. Do you think placements in abortion assessment clinics should be optional or compulsory at medical school? (please circle one answer): Compulsory / Optional / Uncertain

Please feel free to outline any reasons for your response:

6. How much do you agree or disagree with the following statement?

	Strongly disagree	*Disagree*	*Neutral*	*Agree*	*Strongly Agree*
My UCL medical school education on abortion covered everything I need to know					

7. How important do you think it is for medical students to learn about abortion care? (please circle ONE):

Very unimportant / Somewhat unimportant / Neutral / Somewhat important / Very important

8. What are your opinions about the amount of teaching on abortion UCLMS medical students have currently?

	Much too little	*A bit too little*	*About right*	*A bit too much*	*Much too much*
UK law on abortion.					
Doctors' legal and professional right to opt out of providing abortion care.					
Conducting a pregnancy options consultation.					
How to respectfully opt out of abortion care.					
Identifying women who may need counselling before making a decision about abortion.					
Supporting women to make a decision about abortion.					
Referring to an abortion provider.					
Completing the HSA1 (abortion) form.					
What surgical abortion (vacuum aspiration) entails.					
What a surgical abortion (dilatation and evacuation) entails.					

What early medical abortion (<10 weeks gestation) entails.					
What late medical abortion (>10 weeks gestation) entails.					
Abortion-related complications / risks.					
Why women seek abortions.					
Guest speaker: woman who has had an abortion.					
Guest speaker: doctor with a conscientious objection to abortion.					
Clinical exposure to abortion provision.					
Moral arguments for and against abortion.					
Other (please write): ______________________________					

Please comment on any other aspects of abortion care you think there should be more or less teaching on:

Your views on abortion now and in the future

9. Do you personally think it is wrong or not wrong for a woman to have an abortion in the following circumstances?

	Always wrong	*Almost always wrong*	*Wrong only sometimes*	*Not wrong at all*	*Can't choose*
If there is a strong chance of a serious defect in the baby					
If the family has a very low income and cannot afford any more children					

10. What is your overall attitude towards abortion?

Strongly pro-choice	
Moderately pro-choice	

Neither pro-choice nor pro-life	
Moderately pro-life	
Strongly pro-life	
Undecided	
Prefer not to say	

11. In your future career as a doctor, would you be willing to be involved in the following aspects of abortion care (assuming that you had the relevant training and experience)?

**A). Abortions < 12 weeks gestation**	*Yes, in any legally justifiable circumstances*	*Yes, but in specific circumstances only*	*No, under no circumstances*	*Uncertain*
Pregnancy options discussion				
Refer for an abortion				
Complete HSA1 (abortion) form				
Perform medical abortion				
Perform surgical abortion				

**B). Abortions 12-24 weeks gestation**	*Yes, in any legally justifiable circumstances*	*Yes, but in specific circumstances only*	*No, under no circumstances*	*Uncertain*
Pregnancy options discussion				
Refer for an abortion				
Complete HSA1 (abortion) form				
Perform medical abortion				
Perform surgical abortion				

**C). Abortions > 24 weeks gestation**	*Yes, in any legally*	*Yes, but in specific*	*No, under no circumstances*	*Uncertain*

	justifiable circumstances	circumstances only		
Pregnancy options discussion				
Refer for an abortion				
Complete HSA1 (abortion) form				
Perform medical abortion				
Perform surgical abortion				

12. Which specialties are you considering for your future work? Please tick all that apply.

Anaesthestics		*Pathology*	
Care of the Elderly		*Public Health*	
Emergency Medicine		*Psychiatry*	
General Practice		*Radiology*	
Medicine		*Sexual Health*	
Obstetrics and Gynaecology		*Surgery*	
Paediatrics		*Uncertain*	

*Other (please write):*______________________________

About you generally

13. What is your age? __________________

14. What is your gender? ________________________

15. How would you describe your religious beliefs?

*None / Buddhist / Catholic / Hindu / Jewish / Muslim / Protestant / Sikh / Other*______________________________

16. Please feel free to give further comments on abortion education at UCLMS and/or on this

study:

Thank you for completing this anonymous survey

The questionnaire asks some potentially sensitive questions. If completing it raises any issues you would like to talk about with somebody, you can contact Medical Student Support medsch.student-support@ucl.ac.uk or UCL Student Psychological Services: gnandagopak@ucl.ac.uk; 3 Taviton Street, London, WC1E 6BT; hhttp://www.ucl.ac.uk/student-psychological-services/index_home
